# Ugandan Women’s View of the IUD: Generally Favorable but Many Have Misperceptions About Health Risks

**DOI:** 10.9745/GHSP-D-15-00304

**Published:** 2016-08-11

**Authors:** Rogers Twesigye, Peter Buyungo, Henry Kaula, Dennis Buwembo

**Affiliations:** a Programme for Accessible Communication and health Education (PACE), Kampala, Uganda

## Abstract

**Background:**

Between 2001 and 2006, IUD use in Uganda stagnated at 0.2% among women of reproductive age (WRA) ages 15–49. By 2011, IUD use had increased slightly to 0.4%. Recent studies report a significant increase in IUD use to 3.8%, but it is still low. Because the IUD is a little-used method in Uganda, we assessed the acceptability of the IUD by surveying women about their perceptions, attitudes, and beliefs.

**Methods:**

In August and September 2014, we conducted a cross-sectional survey among 1,505 WRA exiting public and private health facilities in Uganda. We collected information on women’s attitudes, knowledge, and beliefs about the IUD, as well as their perceptions about its availability. We classified women’s responses according to a behavior change framework with 3 summary constructs: opportunity (structural factors that influence behavior), ability (skills to perform the behavior), and motivation (self-interest in adopting the behavior). As these 3 types of factors are more favorable to the desired behavior (in this case, use of the IUD), individuals are more likely to perform the behavior. Cross-tabulations compared the percentage results of perceptions and knowledge by key background characteristics.

**Results:**

Most (87.8%) of the surveyed women had heard of the IUD, and nearly two-thirds had a positive attitude toward the method. But a lower percentage (38.6%) had accurate information about the IUD and more than half (51.6%) did not think the method was available in a nearby facility. More than half of the women believed incorrectly that the IUD can damage the womb (57%), that it reduces sexual pleasure (54%), and that it can cause cancer (58%). Current use of family planning or of a modern method specifically was positively associated with awareness and accurate knowledge and beliefs about the IUD. Married women had significantly higher awareness of the IUD than single women, and they had better knowledge and belief scores. The type of facility used for health care services (public, private franchise, or private non-franchise) may also influence acceptance of the IUD.

**Conclusion:**

Interventions to increase the use of IUDs in Uganda should address low availability of the method in facilities, as well as misperceptions and misinformation, especially about the safety of the IUD. Demand promotion should address provider misperceptions in addition to client misperceptions and should include interpersonal communication and the mass media.

## Abstract (precis)

Women in Uganda are aware of the IUD and think it is an effective method, but many think it can damage the womb or make a woman infertile. Addressing public misperceptions through interpersonal communication and the mass media, and provider misperceptions through training, can help to build demand for the IUD in Uganda.

## BACKGROUND

Uganda, with a population of 34.9 million,^1^ has one of the highest population growth rates in the world: 3.3% per year.^2^ Its total fertility rate of 5.8 children per woman in 2015 is also one of the highest in the world, despite a reduction from 6.2 children per woman in 2011.^3^^,^^4^ About 42.6% of recent births were unintended: 27.7% of women wanted a child later, and 14.9% did not want another child.^4^

An almost-equal share of current contraceptive users in the country obtain their methods from the public and private sectors: 47% from public facilities, which provide free family planning services, and 45% from private providers.^3^ Public facilities tend to offer more contraceptive methods than private providers. For example, in 2015 39% of public-sector facilities had IUDs in stock, compared with 7% of private providers, and 48% and 12%, respectively, had implants in stock.^4^

In the past 5 years, the unmet need for family planning in Uganda decreased modestly from 34% of women of reproductive age (WRA) ages 15–49 in 2011 to 30% in 2015. Use of modern contraceptive methods increased from 26% to 32% during the same period, and use of long-acting reversible contraceptives (LARCs)—comprising implants and the intrauterine device (IUD)—increased from 6% to 9% of total modern method use.^4^

**FIGURE 1.**
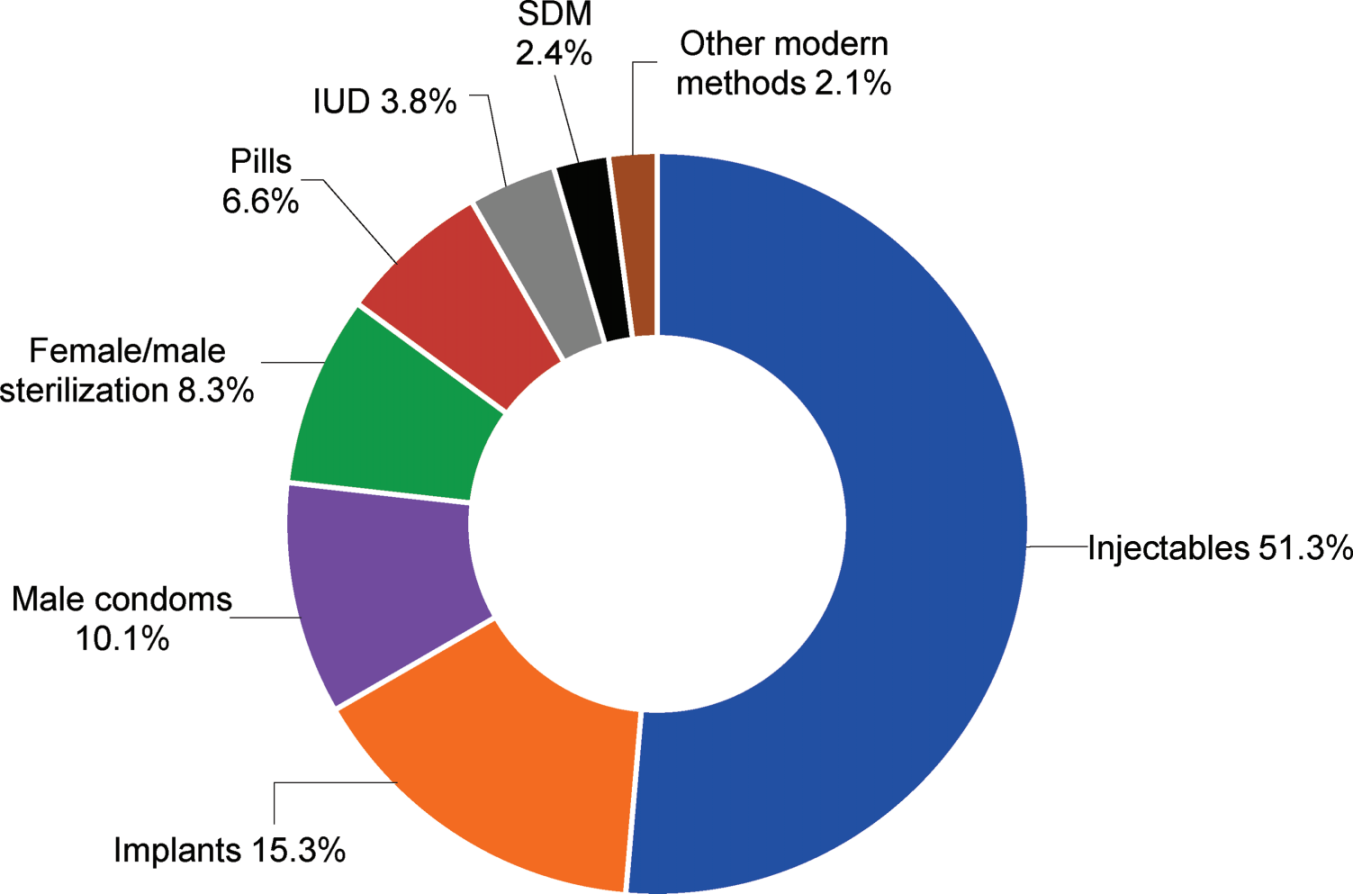
Modern Contraceptive Method Mix Among Married Contraceptive Users, Uganda, 2015 Abbreviations: IUD, intrauterine device; SDM, Standard Days Method. Source: PMA, 2015‐Uganda.^3^

Contraceptive prevalence in Uganda is dominated by injectables, male condoms, and other short-acting methods (Figure 1). Among married contraceptive users, the copper IUD makes up the smallest proportion of the current method mix at 3.8% compared with 6.6% for pills, 8.3% for sterilization (male and female), 10.1% for male condoms, 15.3% for implants, and 51.3% for injectables.^4^ Although the share of the IUD in the method mix is small, use of the method was only 0.4% in 2011, and thus its use has increased dramatically.

Among married contraceptive users, the IUD makes up the smallest proportion of the current method mix at 3.8%.

Because IUDs are a little-used method in Uganda, we wanted to survey women’s knowledge, attitudes, and beliefs about it. We used data from a survey conducted in 2014 among women ages 15–49 years exiting public and private health facilities located in the area of the Women’s Health Project (WHP). This project has been implemented since July 2008 by the Programme for Accessible health Communication and Education (PACE) or Population Services International (PSI) Uganda. A network of ProFam-branded health facilities and partner health facilities carries out WHP. By September 2014, the ProFam franchise had more than 195 facilities in 60 districts in all regions of the country.

WHP aims to increase demand for and access to a wide range of contraceptive methods, with a particular emphasis on increasing access to the IUD, among WRA by improving knowledge and correcting misperceptions related to modern methods through interpersonal communication and the mass media. For interpersonal communication, community-based mobilizers are trained and commissioned to conduct door-to-door sensitization and education of WRA about LARCs and other family planning services. Mass media activities include branding and radio and television advertising. The program also trains providers to insert IUDs and provides insertion kits and subsidized IUDs to franchise facilities.

## METHODS

### Design

**FIGURE 2.**
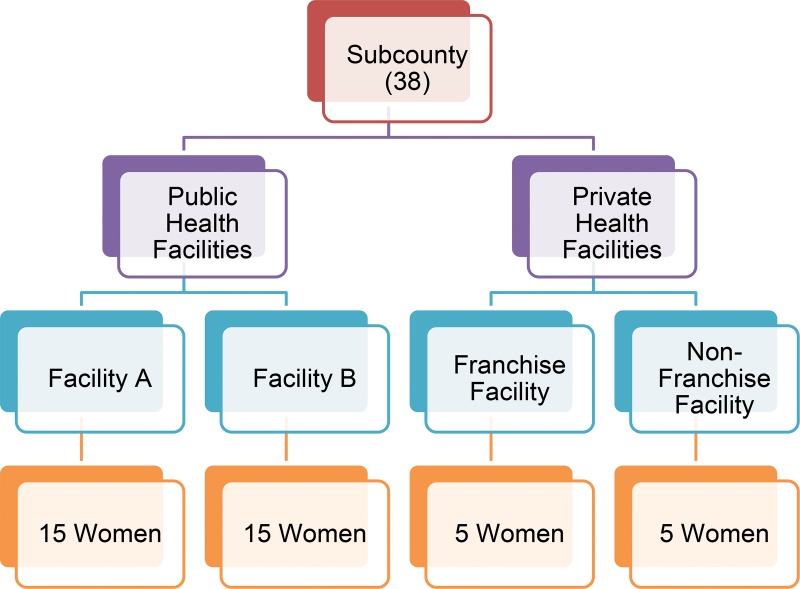
Sampling Procedure: Participants Interviewed at Public, Private, and Pro‐Fam Franchise Facilities in Each of the 38 Selected Subcounties

We carried out the survey in August and September 2014. We used a cross-sectional survey design with structured face-to-face exit interviews, and a multistage cluster sampling to select the study sites at the subcounty and facility level. We drew up a list of all subcounties in 60 project districts with their respective populations and used the probability proportional to population size approach to select 38 subcounties. Four health facilities—2 public and 2 private—were selected in each sampled subcounty using simple random methods from lists of health facilities in the subcounty. For private facilities, we selected 1 franchise facility in the subcounty and 1 non-franchised but private facility (Figure 2).

### Selection of Survey Participants

A total of 1,505 WRA were interviewed when leaving the selected health facilities, irrespective of the services they had received. Interviewers screened all females for eligibility whom they judged to be 15–49 years old. Each selected subcounty contributed 40 WRA toward the overall sample. Public health facilities contributed 30 client exit interviews in each subcounty—15 interviews at each facility. The private health facilities contributed 10 client interviews—5 interviews at the franchise facility and another 5 at the selected non-franchise facility. Analysis for correct knowledge, attitudes, and beliefs was only done among the 87.8% of participants (n = 1,321) who said that they had heard of the IUD, either spontaneously or after prompting.

### Study Instruments and Variables

We collected information on demographics, access to media, fertility history, contraceptive knowledge, and perceptions about family planning in general and about IUDs in particular. We also looked at levels of exposure to communication messages about family planning.

Awareness and knowledge questions were measured using dichotomous variables, whereas perceptions were measured using an evaluation of statements on a 5-point Likert scale, from strongly disagree (1) to strongly agree (5). The questionnaire was designed in English (see supplementary material) and translated into the 6 main languages in the study area.

We classified perceptions according to a behavior change framework adopted by PSI based on a literature review of behavior change theories. This framework has 3 summary constructs: opportunity, ability, and motivation.^5^ Each of the summary constructs comprises different but related factors. As the opportunity, ability, and motivation factors are more favorable to the promoted behavior, individuals are more likely to perform the behavior. The following descriptions were adopted for IUD measurement from the PSI framework.

The PSI behavior change framework has 3 summary constructs: opportunity, ability, and motivation.

- **Opportunity** comprises external or structural factors that influence an individual’s chance to perform a promoted behavior. The opportunity factors measured were awareness and availability. On perceived availability of IUDs, participants were asked to respond to statements such as “IUDs are available in facilities nearby in the community.”
- **Ability** comprises an individual’s skills or proficiencies to perform a promoted behavior. The factor measured under ability was knowledge—whether the individual had correct information about IUDs. Correct information was assessed by 3 statements to which participants had to answer true, false, or don’t know: The IUD can be used while breastfeeding [correct response: true]; Once the IUD is removed, a woman can get pregnant [correct response: true]; and The IUD protects against sexually transmitted infections (STIs) [correct response: false].
- **Motivation** measures a woman’s self-interest in adopting the IUD. Motivation was measured by looking at attitudes, beliefs, and outcome expectations. Attitudes refer to an individual’s assessment of the promoted behavior. Items used to measure attitudes included: The IUD is a good method of family planning; The IUD is a safe family planning method; and The IUD is an effective family planning method. Women chose answers from the 5-point Likert scale. Beliefs are perceptions about a promoted behavior that may or may not be true. Items were measured on the 5-point Likert scale and included: IUDs can result in cancer; IUDs can damage the womb; and Prolonged use of IUDs can cause infertility.

### Data Analysis

We used SPSS version 18 to analyze the data. Correct knowledge was computed by creating a new variable for participants who gave correct answers for all 3 knowledge questions. For the scaled responses, we conducted factor analysis to load similar statements together that resulted in 2 components—attitudes and beliefs/myths about the IUD. We used Cronbach’s alpha to calculate internal reliability and consistency of the 2 created components (Table 1). Composite scores based on a scale of 1–5 were thereafter computed for each created component. A higher score related to a positive attitude (acceptable) and a lower score to a negative attitude (unacceptable). The reverse was true for the score on the second component related to myths about the IUD; a lower score in this case related to a positive attitude. Cross-tabulations compared the percentage results of perceptions and knowledge by key population characteristics, and we conducted UNIANOVA analysis to compare mean scores for the 2 created components and key characteristics and to test the significance of observed differences.

**TABLE 1.** Reliability Analysis Using Cronbach’s Alpha for Attitudes and Beliefs About the IUD

	Cronbach’s alpha
Attitudes	0.862
The IUD is a good method of family planning.	
IUDs are safe.	
The IUD is an effective contraceptive method for women like me.	
Beliefs	0.880
Using IUDs can result in cancer.	
IUDs can damage the womb.	
Prolonged use of IUDs can cause infertility.	

Abbreviations: IUD, intrauterine device.

### Ethical Procedures

The study was approved by the Mildmay Uganda Research Ethics Committee (MUREC) and registered with the Uganda National Council for Science and Technology (UNCST).

## RESULTS

### Characteristics of the Study Participants

The majority (85.9%) of the survey participants were 34 years or less, and 70.3% were either married or cohabiting (Table 2). Slightly over half (52.3%) of the participants had either no education or only a primary education; the rest had a secondary education or higher. More than three-quarters (81.0%) were Christians (32.1% Catholics, 29.7% Protestant, and 19.2% Pentecostal), and 18.9% were Muslims. Half (50.8%) of the participants reported that they regularly or at times read newspapers. A similar proportion regularly or at times watched television, whereas almost all (92.4%) listened to the radio sometime every month.

**TABLE 2.** Characteristics of Survey Participants (N = 1,505)

Characteristic	%
Age group	
15–24	48.2
25–34	37.7
35–49	14.1
Marital status	
Not married	29.7
Married/cohabiting	70.3
Education	
No formal education	7.7
Primary	44.6
Secondary	38.5
Above secondary	9.2
Religion	
Catholic	32.1
Protestant	29.7
Pentecostal	19.2
Muslim	18.9
Access to media	
Reads newspapers	50.8
Listens to radio	92.4
Watches television	56.7
Parity	
Ever given birth	81.2
Never given birth	18.8
No. of living children	
0	18.8
1–2	40.6
3–4	23.5
5 or more	17.1
Contraceptive use	
Any method	53.0
Modern method	44.1

More than three-quarters of the participants (81.2%) had ever given birth in their lifetime (Table 2); 40.6% had given birth to 1 or 2 children, about one-quarter (23.5%) had 3–4 children, and 17.1% had given birth to 5 or more children. Just over half (53.0%) of the participants (exclusive of those who were pregnant) were using some form of contraception to delay or avoid pregnancy, and 44.1% were using a modern contraceptive method.

### Opportunity: Awareness of the IUD

The majority (87.8%) of the participants had heard of the IUD as a contraceptive method. Awareness of the IUD varied by marital status, age group, current use of family planning, and TV viewership (Table 3). For example, significantly more married women had heard of the IUD than unmarried women (90.8 vs. 80.5%, respectively; *P*<.001). Similarly, significantly more women using any form of contraception had ever heard of the IUD than those not using a contraceptive (91.5% vs. 82.5%, respectively; *P*<.001). The trend was similar for women using modern contraceptives in comparison with those using traditional methods (92.6% vs. 86.1%, respectively; *P* = .001). No statistically significant differences were observed in awareness of the IUD and parity, whether one listens to the radio, or the type of facility used for health care services.

Most surveyed women had heard of the IUD.

**TABLE 3.** Opportunity and Ability Factors (%) Related to IUDs Among Survey Participants by Background Characteristics

	Opportunity				Ability	
Characteristic	Heard of IUDs (N=1,505)	*P* Value	Perception of IUD Availability (n=1,321)^a^	*P* Value	Correct IUD Knowledge (n=1,321)^b^	*P* Value
Marital status		**<.001**		.45		**.005**
Married/cohabiting	90.8		49.0		40.9	
Not married	80.5		46.7		32.5	
Age		**<.001**		**.02**		**.01**
15–24	84.0		45.0		34.4	
25–34	92.0		53.0		41.8	
35–49	89.2		46.0		43.8	
Parity		.92		.85		.07
1–2	90.5		48.8		37.4	
3–4	90.9		50.8		41.7	
5 or more	89.9		50.0		46.1	
Uses family planning		**<.001**		.97		**.006**
No	82.5		48.9		33.7	
Yes	91.5		49.0		41.9	
Type of family planning		**.001**		**.05**		**.03**
Traditional	86.1		43.3		35.6	
Modern	92.6		50.3		43.3	
Listens to radio		.13		.30		**.006**
Not at all	85.3		72.5		25.3	
Usually	88.1		78.7		39.6	
Watches TV		**.01**		.70		.83
Not at all	85.3		77.7		38.9	
Usually	89.7		78.8		38.4	
Type of facility used for health care services		.29		**.008**		.30
Public	86.5		48.6		36.5	
Private, non-franchise	89.5		45.6		40.3	
Private, franchise	87.9		58.6		41.4	
Total	87.8		48.4		38.6	

Abbreviation: IUD, intrauterine device.

Significant *P* values ≤ .05 are shown in boldface.

a Percentage of women who had heard of IUDs and who responded positively to the statement, “IUDs are always available in the community and in a facility nearby.”

b Percentage of women who had heard of IUDs and who knew the correct answers to the following statements: “IUDs can be used while breastfeeding” (correct answer: yes); “Once removed, a woman can get pregnant” (correct answer: yes); and “IUDs protect against sexually transmitted infections” (correct answer: no).

### Opportunity: Perceived Availability of IUDs

Overall, 48.4% of participants perceived IUDs as available in a facility nearby in the community. Perceived availability of IUDs varied by use of modern contraception, age group, and source of health services. Most notably, significantly more women using modern contraceptives than those using traditional methods perceived IUDs as available in a nearby facility (50.3% vs. 43.3%, respectively; *P* = .05). No statistically significant differences were observed on perceived availability of IUDs and marital status, parity, use of any family planning method, radio listenership, or TV viewership.

### Ability: Correct Knowledge About IUDs

Overall, 38.6% of the women who had heard of the IUD knew that it can be used during breastfeeding, that a woman can get pregnant once the IUD is removed, and that the IUD does not protect against STIs. Correct information about the IUD varied by marital status, age group, current use of family planning, and radio listenership (Table 3). No statistically significant differences were observed in correct knowledge about IUDs and parity, TV viewership, or the type of facility used for health care services.

### Motivation: Attitude Toward IUDs

The study results showed that nearly two-thirds of the women believed that IUDs are safe (60.3%) and are a good method of family planning (63.7%). In addition, 59.0% reported that IUDs are an effective family planning method, while a notable percentage (45.8%) would recommend an IUD to a friend. Women scored the IUD above average (3.6 out of 5) as a good, safe, and effective family planning method (Table 4). No background characteristic showed significant variations on attitude toward the IUD.

**TABLE 4.** Motivation Factors Related to IUDs Among Survey Participants by Background Characteristics (N = 1,505)

Characteristic	Mean Attitude Score^a^	*P* Value	Mean Beliefs Score^b^	*P* Value
Marital status		.62		**<.001**
Married/cohabiting	3.6		3.0	
Not married	3.6		3.5	
Age group		.09		**.04**
15–24	3.5		3.2	
25–34	3.9		3.0	
35–49	3.5		3.4	
Parity		.64		.86
1–2	3.6		3.2	
3–4	3.6		3.1	
5 or more	3.7		3.0	
Uses family planning		.35		**.002**
No	3.4		3.4	
Yes	3.6		3.0	
Type of family planning		.44		.08
Traditional	3.6		3.2	
Modern	3.6		3.0	
Listens to radio		.48		.89
Not at all	3.4		3.1	
Usually	3.6		3.2	
Watches TV		.71		.38
Not at all	3.6		3.2	
Usually	3.6		3.1	
Facility type		.25		**.003**
Public	3.6		3.2	
Private, non-franchise	3.5		3.3	
Private, franchise	3.7		2.8	
Overall score	3.6		3.6	

Abbreviation: IUD, intrauterine device.

Significant *P* values ≤ .05 are shown in boldface.

a Women’s scores out of a total 5 for the following 3 statements: The IUD is a good family planning method; The IUD is a safe family planning method; and The IUD is an effective family planning method.

b Women’s scores out of a total 5 for the following 3 statements: The IUD can result in cancer; The IUD can damage the womb; and Prolonged use of IUDs can cause infertility.

### Motivation: Beliefs About IUDs

Overall, women seemed to agree with or were not sure about the incorrect beliefs about the IUD. More than half (57%) of the women believed IUDs can damage the womb and a similar percentage (54%) thought IUDs reduce sexual pleasure. More than half (58%) also believed that the IUD can cause cancer while close to half (48%) believed that prolonged IUD use can cause infertility. On average, women scored the IUD 3.6 out of 5 in relation to causing cancer, damaging the womb, and causing infertility.

Nearly two-thirds of surveyed women believed that IUDs are safe, but many surveyed women had misconceptions about the method.

Beliefs about IUDs were found to vary by marital status, age group, current use of family planning, and source of health services (Table 4). Married women, for example, scored significantly lower than unmarried women on the incorrect beliefs about IUDs (3.0 vs. 3.5; *P*<.001). This implies that married women refuted incorrect beliefs about IUDs more than their counterparts and therefore were more likely to accept the IUD. Notably, women at the project franchise facilities were more accepting of the IUD compared with those at public and other private facilities. No statistically significant differences were observed in incorrect beliefs and number of children, use of modern contraceptives, and TV viewership or radio listenership.

## DISCUSSION

Despite the low use of the IUD in Uganda, a high percentage of women surveyed in this study—nearly 90%—have heard of the IUD. By comparison, in the 2011 Uganda Demographic and Health Survey, 70.2% of women said that they had heard of the IUD.^3^ The surveyed women in this study generally have a positive attitude toward the IUD as a safe and effective family planning method, but the majority have inaccurate knowledge about the method, for example, about return to fertility after IUD removal, and the majority also believe in myths surrounding the IUD. For example, over 40% of the participants had incorrect information about the IUD in relation to breastfeeding, return to fertility, and protection against STIs. Participants also believed incorrectly that IUDs cause cancer and damage the womb.

Availability of IUDs is limited by the lack of skilled providers and the lack of equipment. Over half of the survey respondents perceived a lack of availability of IUDs in nearby facilities. This perception is supported by findings from other surveys. For example, in a 2014 survey of private-sector providers, over 70% of the providers had ever had training in IUD insertion, but only 4% of franchise providers and 13% of non-franchise providers had comprehensive training, including lectures, practice on models, and performing an insertion and removal on a patient under observation.^6^ Furthermore, only 30% of the providers correctly identified 3 of 5 side effects of the IUD.^6^ Providers are a key source of information for women seeking family planning, and a knowledge gap on their side results in a knowledge gap among women, which affects acceptance of a particular family planning method.

Over half of the surveyed women did not think the IUD was available in a nearby facility.

In our study, several factors emerged as having an influence on awareness, knowledge, and beliefs about the IUD. Current use of any family planning method or of a modern method specifically was positively associated with awareness and accurate knowledge and beliefs about the IUD. Women using contraceptives are more likely to be exposed to information about the IUD at a facility or from a health care provider than nonusers. In addition, married women had significantly higher awareness of the IUD than single women and had better knowledge and belief scores. This might be attributed to the fact that married women have already had children and therefore are in need of limiting births or spacing for a longer period. TV viewership seemed to play a more significant role in improving awareness about the IUD, whereas radio played a more significant role in women having correct knowledge about the method. Finally, the type of facility used for health care services may also influence acceptance of the IUD as a contraceptive method. Women at franchise facilities believed less in IUD-related myths than women at public facilities or private but non-franchise clinics. Women at franchise clinics also perceived the IUD as being available in the community. This might be attributed to the efforts to increase IUD use by PACE through its Women’s Health Project.

### Study Limitations

Conducting the study in project implementation areas may have led to some bias since the ProFam franchise is in only 60 of 112 districts. However, we believe that this bias was reduced by the nationwide promotion of the IUD and the use of a random sample of non-franchise facilities and participants. Although women were selected randomly, only WRA who visited facilities on days when research teams were visiting had the chance to participate in the study. The study may also suffer from a seasonality effect since data were collected over a short period of time.

## CONCLUSION

Use of the IUD in Uganda is increasing rapidly but is still low. To further increase access to and use of the IUD, couples need accurate information about the benefits and risks of the IUD. This information can come through interpersonal communication, from trained providers, and in the mass media. To protect women’s health in Uganda, promoting demand for this safe and effective contraceptive method, in the context of wide method choice, should be a priority.

To protect women’s health and slow Uganda’s rapid population growth rate, promoting demand for the IUD, in the context of wide method choice, should be a priority.

## Supplementary Material

supplementary material

## Notes

**Peer Reviewed**
