# Supplementary material for: Ugandan Women’s View of the IUD: Generally Favorable but Many Have Misperceptions About Health Risks
Source: Glob Health Sci Pract. 2016 Aug 11;4(Suppl 2):S73–82. doi: 10.9745/GHSP-D-15-00304 (PMC4990164; doi:10.9745/GHSP-D-15-00304)
Supplement: supplementary material [file GHSP-D-15-00304_index.html]

Supplement to Ugandan Women’s View of the IUD: Generally Favorable but Many Have Misperceptions About Health Risks | Global Health: Science and Practice

## GHSP-D-15-00304 Supplementary Material

Twesigye et al. doi: 10.9745/GHSP-D-15-00304

- supplementary material - Twesigye et al. doi: 10.9745/GHSP-D-15-00304
