# Supplementary material for: Ugandan Women’s View of the IUD: Generally Favorable but Many Have Misperceptions About Health Risks
Source: Glob Health Sci Pract. 2016 Aug 11;4(Suppl 2):S73–82. doi: 10.9745/GHSP-D-15-00304 (PMC4990164; doi:10.9745/GHSP-D-15-00304)
Supplement: supplementary material [file 15-00304-Twesigye-Supplementary-material.docx]

**Program for Accessible health Communication and Education (PACE)**

**FAMILY PLANNING QUESTIONNAIRE for WOMEN, Uganda, 2014 (ENGLISH VERSION)**

**Section 1: Identification and Screening Questions**

# N.B: Fill in all the required information before starting the interview

| **Interview ID:** Interviewer-District-Sub county-Questionnaire ID [__\|__] - [__\|__]-[__\|__\|__]-[__\|__\|__] | |
| --- | --- |
| **1.1. Today’s date** DD-MM-YYYY: [__\|__]-[__\|__]-[_2_\|_0_\|_1_\|_4_] | |
| **1.2. Interviewer’s name:** | 1.2a. Interviewer code [__\|__] |
| **1.3. District:** | 1.3a. District code [__\|__] |
| **1.4. Sub-County/Division:** | 1.4a. Sub-County code [__\|__\|__] |
| **1.5. Name of Health Facility:** | |
| **1.6. Health facility Category:** | Public --------1 Private --------0 |
| **1.7. Location** **(Rural/Urban):** | Urban--------1 Rural---------0 |

**Screening Questions**

| **NO** | **QUE QUESTION** | **RESPONSE** | **CODE** | **SKIP** |
| --- | --- | --- | --- | --- |
| **S1** | Do you usually sleep within this sub county/Division? ***(For at least four nights a week)*** | Yes  No | 1  0 |  |
| **S2** | How old are you in complete years? | 15-49 years  Other age bracket | 1  0 | **If 15-49 yrs, Seek full consent** |
| **S3** | Do you allow to be interviewed for some minutes? | Yes  No | 1  0 | **If yes, Provide information sheet and start Interview** |

| **Interview result**  1 = Completed interview  2 = Woman not eligible  3 = Interview interrupted  4 = Refused ***(Comment)***  96 = Other *(specify)* [____________] | | [____] | [____] | [____] |
| --- | --- | --- | --- | --- |
| Time started:  Time completed: | ***(use 24hr clock N/A = 00:00)*** | [___\|___:___\|___]  [___\|___:___\|___] | [___\|___:___\|___]  [___\|___:___\|___] | [___\|___:___\|___]  [___\|___:___\|___] |
| **Comments *(If the WRA refuses to participate, provide reason for refusal.)*** | | | | |

**SECTION II: DEMOGRAPHIC INFORMATION, ACCESS TO MEDIA, FERTILITY HISTORY, CONTRACEPTIVE KNOWLEDGE AND USE**

COMPLETE THIS SECTION FOR EVERY ELIGIBLE WOMAN OF REPRODUCTIVE AGE (WRA)

**Module I: Demographic Information**

| **Interviewer say: Now I would like to ask you some questions about you and your background. Feel free to answer these questions as they are to help me learn from you** | | | |  |
| --- | --- | --- | --- | --- |
| **Q201** | How old were you at your last birthday? | **Age in Complete years** |  |  |
| **Q202** | What is your **highest** level of education attained?  ***DON’T READ ANSWERS, SELECT ONE RESPONSE*** | None  Primary  ‘O’ Level  ‘A’ Level  Tertiary  University  Don’t Know | 1  2  3  4  5  6  98 | **Q204** |
| **Q203** | Do you read a newspaper or magazine almost every day, at least once a week, less than once a week/rarely, or not at all? | Almost every day  At least once a week  Less than once a week/rarely  Not at all | 1  2  3  4 |  |
| **Q204** | Do you listen to the radio almost every day, at least once a week, less than once a week/rarely, or not at all? | Almost every day  At least once a week  Less than once a week/rarely  Not at all | 1  2  3  4 |  |
| **Q205** | Do you watch television almost every day, at least once a week, less than once a week/rarely, or not at all? | Almost every day  At least once a week  Less than once a week/rarely  Not at all | 1  2  3  4 |  |
| **Q206** | What is your religion?  ***DON’T READ ANSWERS, SELECT ONE RESPONSE*** | Catholic  Protestant  Muslim  Pentecostal  SDA  Other (**specify**)_________ | 1  2  3  4  5  96 |  |
| **Q207** | What ethnic group/tribe do you belong to?  ***DON’T READ ANSWERS, SELECT ONE RESPONSE*** | Muganda  Musoga  Munyankole/Mukiiga  Muteso  Munyoro-Mutooro  Acholi  Langi  Lugbara  Other (**specify**)__________ | 1  2  3  4  5  6  7  8  96 |  |
| **Q208** | Are you currently married or living together with a man as if married?    ***DON’T READ ANSWERS, SELECT ONE RESPONSE*** | Yes, currently Married  Yes, living with a man  No, not married | 1  2  3 | **Q210**  **Q210** |
| **Q209** | Have you ever been married or lived together with a man as if married? | Yes, formerly married  Yes, lived with a man  No, never | 1  2  3 |  |

**Module II: Fertility history and Preferences**

**Interviewer say: *Now I would like to ask about all the births you have had during your life.***

| **NO** | **QUESTION** | **RESPONSES** | **CODE** | **SKIP** |
| --- | --- | --- | --- | --- |
| **210** | Have you ever given birth? | Yes  No | 1  0 | **Q214** |
| **211** | How many living biological children do you have?  **IF NONE, RECORD 00**  **Since we are looking for live births, then edit the question to remove “living children” and put live births** |  |  |  |
|  | ***CHECK ANSWER TO Q211***  ***The check is on Q211*** | **IF Total live births >0**  **IF Total live births=0** |  | **Q212**  **Q214** |
| **212** | How many months ago did you give birth to your last baby?  ***RECORD IN MONTHS*** | **Months** |  |  |
| **213** | Are you currently breastfeeding?  ***CHECK ANSWER TO Q312. IF THE RESPONDENT GAVE BIRTH OVER 36 MONTHS AGO, SELECT N/A.*** | Yes  No  N/A | 1  0  98 |  |
| **215** | When you (last) got pregnant, did you want to get pregnant at that time?  ***IF RESPONDENT HAS NEVER BEEN PREGNANT, MARK N/A*** | Yes  No  N/A | 1  0  98 | **Q217**  **Q217** |
| **216** | Did you want to have a baby later on or did you not want any (more) children? | Later  More | 1  2 |  |

**Module III: Contraceptive Knowledge**

| **Interviewer say: In this section, I would like to talk about family planning - the various ways or methods that a one can use to delay or avoid a pregnancy. I would like to know which family planning methods do you know or have you heard about** | | | | |
| --- | --- | --- | --- | --- |
| **217** | Have you ever heard about methods that can be used to avoid pregnancies? | Yes  No | 1  0 |  |
| **218** | ***Use picture cards for FP methods not known***  (LF) What family planning methods do you know or have heard of?  **FOR METHODS NOT MENTIONED SPONTANEOUSLY ask;** Have you heard of (METHOD) ***Reading the Name and Description of each Method Not Recognized Spontaneously Circle* 2 *if Recognized*** | I know or heard Spontaneous | I’ve heard or know Prompted | Don’t know |
|  | **Pills** ( A woman can take a pill a day to avoid getting pregnant) | 1 | 2 | 0 |
|  | **Injectables** ( Women can have an injection by a health provider which stops them from becoming pregnant for two or three months) | 1 | 2 | 0 |
|  | **Emergency contraception** ( Pills taken up to five days after sex to prevent a woman getting pregnant) | 1 | 2 | 0 |
|  | **Male condoms** ( Men can put a rubber sheath over their penis before sex) | 1 | 2 | 0 |
|  | **Female condoms** (Women can place a rubber sheath in their vagina before sexual intercourse) | 1 | 2 | 0 |
|  | **IUD** (Women can have a coil placed inside the womb by a doctor or FP provider which protects against pregnancy for up to 12 years) | 1 | 2 | 0 |
|  | **Implants** (Women can have small plastic rods placed in their upper arm by a doctor or nurse and this can prevent pregnancy for up to 5 years) | 1 | 2 | 0 |
|  | **Male Sterilization** (Men can have an operation to avoid having any more children) | 1 | 2 | 0 |
|  | **Female sterilization** (Women can have an operation to avoid having any more children) | 1 | 2 | 0 |
|  | **Lactational amenorrhea** (Up to 6 months after child birth a woman can use a method that requires that she breastfeeds day and night and her menses has not returned as a form of Family planning) | 1 | 2 | 0 |
|  | **WITHDRAWAL** (Men can be careful and pull out just before ejaculation) | 1 | 2 | 0 |
|  | Periodic abstinence - **Calendar Method/Beads** | 1 | 2 | 0 |
|  | Other traditional method (specify)…………………… | 1 | 2 | 0 |

**Module IV: Contraceptive use**

| **NO** | | | **QUESTION** | | **RESPONSES** | | **CODE** | **SKIP** | | | | | |
| --- | --- | --- | --- | --- | --- | --- | --- | --- | --- | --- | --- | --- | --- |
| **219** | | | Are you pregnant now? | | Yes  No  Not sure/Don’t know | 1  0  98 | | **If no skip to Q222** | | | | |  |
| **220** | | | After the child you are expecting now, would you like to have another child or any more children? | | Have a child or another child  No more/None  Undecided/Don’t know | | 1  2  98 | **If no/DK Q224** | | | | | |
| **221** | | | After how many months would you want to have another child?  Maybe we should consider asking Q225 to WRA who are not pregnant but not using any FP method. It will help reduce the confusion, the way it is now, we may need an instruction telling interviewers not ask the pregnant WRA but just tick appropriately. | | **MONTHS**  After Marriage  Don’t Know  Other (Specify) _________________ | | 95  98  97 | **Any response go to 225** | | | | | |
| **222** | | | Would you like to have a child in the future? | | Yes  No  Undecided/Don’t know | | 1  2  98 | **If no/DK go to Q224** | | | | | |
| **223** | | | After how many months would you want to have a child? | | **MONTHS** | | 93  95  98  97 |  | | | | | |
|  |  |  |  |  | Soon/Now  After Marriage  Don’t Know  Other (Specify) _________________ | |  |  |  |  |  |  |  |
| **224** | | | Are you currently doing something or using any method to delay or avoid getting pregnant?  I think the WRA who is currently using, needs not be asked 226, it becomes confusing, should just go to 229 – it is one who is not pregnant and not currently using anything that we should check to see whether has ever used. | | Yes  No  N/A | | 1  0  3 | **If yes go to 226** | | | | | |
| **225** | | | Why are you not using any family planning methods? | | **Responses** | | **Y** | **N** | | | **N/A** | |  |
|  |  |  |  |  | 1. Not Married | | 1 | 0 | | | 98 | |  |
|  |  |  |  |  | ***Fertility-related reasons*** | | | | | | | |  |
|  |  |  |  |  | 1. Currently pregnant | | 1 | 0 | | | 98 | |  |
|  |  |  |  |  | 1. Not having sex | | 1 | 0 | | | 98 | |  |
|  |  |  |  |  | 1. Infrequent sex | | 1 | 0 | | | 98 | |  |
|  |  |  |  |  | 1. Menopausal/hysterectomy | | 1 | 0 | | | 98 | |  |
|  |  |  |  |  | 1. Can’t get pregnant | | 1 | 0 | | | 98 | |  |
|  |  |  |  |  | 1. Not menstruated since last birth | | 1 | 0 | | | 98 | |  |
|  |  |  |  |  | 1. Breast feeding | | 1 | 0 | | | 98 | |  |
|  |  |  |  |  | 1. Up to God/fatalistic | | 1 | 0 | | | 98 | |  |
|  |  |  |  |  | ***Opposition to use reasons*** | | | | | | | |  |
|  |  |  |  |  | 1. Respondent opposed | | 1 | 0 | | | 98 | |  |
|  |  |  |  |  | 1. Husband/partner opposed | | 1 | 0 | | | 98 | |  |
|  |  |  |  |  | 1. Mother-in-law opposed | | 1 | 0 | | | 98 | |  |
|  |  |  |  |  | 1. Other opposed | | 1 | 0 | | | 98 | |  |
|  |  |  |  |  | 1. Religious opposed | | 1 | 0 | | | 98 | |  |
|  |  |  |  |  | ***Knowledge reasons*** | | | | | | | |  |
|  |  |  |  |  | 1. Knows no method | | 1 | | | 0 | | 98 |  |
|  |  |  |  |  | 1. Knows no source | | 1 | | | 0 | | 98 |  |
|  |  |  |  |  | ***Method-related reasons*** | | | | | | | |  |
|  |  |  |  |  | 1. Side effects/health concerns | | 1 | | 0 | | | 98 |  |
|  |  |  |  |  | 1. Lack of access/too far | | 1 | | 0 | | | 98 |  |
|  |  |  |  |  | 1. Costs too much | | 1 | | 0 | | | 98 |  |
|  |  |  |  |  | 1. Preferred method not available | | 1 | | 0 | | | 98 |  |
|  |  |  |  |  | 1. No method available | | 1 | | 0 | | | 98 |  |
|  |  |  |  |  | 1. Inconvenient to use | | 1 | | 0 | | | 98 |  |
|  |  |  |  |  | 1. Interferes with body’s normal process | | 1 | | 0 | | | 98 |  |
|  |  |  |  |  | 1. Don’t know | | 1 | | 0 | | | 98 |  |
|  |  |  |  |  | 1. Not Applicable | | 1 | | 0 | | | 98 |  |
|  |  |  |  |  | 1. Others specify | | 1 | | 0 | | | 98 |  |
| **226** | | | Have you used anything in the recent past or tried in any way to delay or avoid getting pregnant?  A WRA who has not used any FP in the recent past, cannot answer Q228, because we don’t know whether she has ever used FP in her life, maybe she skips to Q235 | | Yes  No | | 1  0 | | **Skip to 228** | | | | |
| **227** | | | Which method(s) have you used?  ***READ ALL ANSWERS AND ALLOW FOR MULTIPLE RESPONES*** | | Female Sterilization  Male Sterilization  IUD  Injectables  Implants  Pills  Emergency Contraception  Male condom  Female condom  Diaphragm  Foam/Jelly  Cycle beads  Lactational Amen method  Rhythm method  Withdrawal  Other **(Specify)** ________________ | | 1  2  3  4  5  6  7  8  9  10  11  12  13  14  15  97 | |  | | | | |
| **228** | | | How old were you when you first used a method to delay or avoid getting pregnant?  There will be some WRA who are not using any FP currently answering this question – if they used FP more than 1 year ago, do we want to know where they got it, cost, etc? if yes, we put skip to Q 230, if not then to Q 235 | | Age in complete years | |  | |  | | | | |
| **229** | | | Which method(s) are you currently using?  ***IF CURRENTLY USING MORE THAN ONE METHOD, CIRCLE ALL THAT APPLY*** | | 1. Currently Pregnant 2. Not using any FP method 3. Female Sterilization 4. Male Sterilization 5. IUD 6. Injectables 7. Implants 8. Pills 9. Emergency Contraception 10. Male condom 11. Female condom 12. Diaphragm 13. Foam/Jelly 14. Cycle beads 15. Lactational Amen method 16. Rhythm method 17. Withdrawal 18. Other **(Specify)** _____________ | | 1  2  3  4  5  6  7  8  9  10  11  12  13  14  15  16  97 | | **If Currently pregnant go to 234**  **If Not using FP go to Q235** | | | | |
| **230** | | | Where did you receive your INSERT CURRENT METHOD? | | ***PUBLIC SECTOR***  "Government Health Centre  Mobile public clinic or Out reach  ***PRIVATE MEDICAL SECTOR***  Private clinic /hospital  Pharmacy/Drug shop  Franchise/Network clinic  Mobile private clinic or out reach  Fieldworker e.g VHT  ***OTHER SOURCES***  Shop  Friend/Relative  NGO clinic  Not Applicable  Other **(Specify)** _____________ | | 1  2  3  4  5  6  7  8  9  10  11  96 | | **If not applicable go to Q232** | | | | |
| **231** | | | How much did you pay for *INSERT CURRENT METHOD*? | | **Insert Amount in Uganda Shillings** | |  | |  | | | | |
| **232** | | | How long ago did you start using INSERT CURRENT METHOD for preventing pregnancy?  ***RECORD IN MONTHS, LESS THAN ONE MONTH, RECORD "00".*** | | **MONTHS**  I think we should move Q 232, 233, & 234 to come immediately after Q229 – all these apply to only WRA who are currently using an FP method.  When we do this shift, it enables us ask the WRA who is not using FP now to move from Q228 to current Q230 | |  | |  | | | | |
| **233** | | | Are you satisfied with your main current family planning method? | | Yes  No  No Opinion | | 1  0 | | **If yes skip to 235** | | | | |
| **234** | | | **If no:** Why are you not satisfied with your current method?  **MULTIPLE ANSWERS POSSIBLE** | | Discomforts  Irregularities of my menstrual periods  Side effects  Cost  Inconvenience of using it  My partner doesn’t support it  Fear about fertility  Other(sp)____________ | | 1  2  3  4  5  6  7  96 | |  | | | | |
|  | | | **228** | |  | |  | |  | | | | |
| **235** | Do you think you will use/continue using a contraceptive method in the next 12 months? | | | | Yes  No  Don’t Know/Unsure | 1  0  98 | | **If no/DK go to 237** | | | | |  |
| **236** | **(LF)** What method do you think you will use in the next 12 months?  (**One answer**) | | | | Female Sterilization  Male Sterilization  IUD  Injectables  Implants  Pills  Emergency Contraception  Male condom  Female condom  Diaphragm  Foam/Jelly  Cycle beads  Lactational Amen method  Rhythm method  Withdrawal  Other **(Specify)** ________________ | 1  2  3  4  5  6  7  8  9  10  11  12  13  14  15  96 | |  | | | | |  |
| **237** | Do you think you will use/continue using a contraceptive method to delay or avoid pregnancy any time after 12 months? | | | | Yes  No  Don’t Know/Unsure | 1  0  98 | |  | | | | |  |
| **238** | | | Overall, What method(s) of family planning would you prefer to use?  (LF)  **MULTIPLE ANSWERS POSSIBLE** | | Pills  Injectables  Emergency contraception  Male condoms  Female condoms  Diaphragm  Foams/Gel  IUD  Implants  Male sterilization  Female sterilization  Lactational amenorrhoea  Periodic abstinence  Withdrawal  Other traditional 1..................  Other traditional 2.................... | | 01  02  03  04  05  06  07  08  09  10  11  12  13  14  15  16 |  | | | | | |

**SECTION III: PERCEPTIONS ABOUT FAMILY PLANNING AMONG WOMEN OF REPRODUCTIVE**

COMPLETE THIS SECTION FOR EVERY ELIGIBLE WOMAN OF REPRODUCTIVE AGE (WRA)

| ***Interviewer say:*** Now I would like to ask about what factors make it easy or difficult to use contraception. For the following statements, please answer 'yes' if you agree, or 'no' if you disagree. | | | | |
| --- | --- | --- | --- | --- |
| **OPPORTUNITY** | | | | |
| **Availability** | | **Yes** | **No** | **DK** |
| 301 | I know of a place where to get contraceptives in my community. | 1 | 0 | 9 |
| 302 | Profam clinics are available nearby | 1 | 0 | 9 |
| 303 | IUCDs are available at a facility nearby | 1 | 0 | 9 |
| 304 | Profam clinics have any family planning method you may want | 1 | 0 | 9 |
| 305 | IUCDs are always available in public health facilities | 1 | 0 | 9 |
| 306 | IUCDs are always available at Profam clinics | 1 | 0 | 9 |
| 307 | IUCDs are always available in Blue star clinics | 1 | 0 | 9 |
| 308 | Implants are always available at Profam clinics | 1 | 0 | 9 |
| **Affordability** | |  |  |  |
| 309 | IUCDs are affordable in my community | 1 | 0 | 9 |
| 310 | Implants are affordable in my community | 1 | 0 | 9 |

| ***Interviewer say:*** For the following statements, please respond from a scale of 1 to 5 where 1 means you strongly disagree and 5 means you strongly agree | | | | | | | | | | | | |
| --- | --- | --- | --- | --- | --- | --- | --- | --- | --- | --- | --- | --- |
| **OPPORTUNITY** | | | | | | | | | | | | |
|  | | **Strongly Disagree** | | **Somewhat**  **Disagree** | | **Neutral** | | **Somewhat Agree** | | **Strongly Agree** | | **DK** |
| **Brand Appeal** | |  | |  | |  | |  | |  | |  |
| 311 | I have heard about Profam clinics | 1 | | 2 | | 3 | | 4 | | 5 | | 9 |
| 312 | Services from Profam clinics are of high quality | 1 | | 2 | | 3 | | 4 | | 5 | | 9 |
| 313 | FP methods provided from Profam clinics are safe | 1 | | 2 | | 3 | | 4 | | 5 | | 9 |
| 314 | Profam clinics are for women like me | 1 | | 2 | | 3 | | 4 | | 5 | | 9 |
| **Brand Attributes** | |  | |  | |  | |  | |  | |  |
| 315 | Profam clinics are for responsible mothers | 1 | | 2 | | 3 | | 4 | | 5 | | 9 |
| 316 | Profam clinics are for the rich | 1 | | 2 | | 3 | | 4 | | 5 | | 9 |
| 317 | Profam clinics have the most caring health workers | 1 | | 2 | | 3 | | 4 | | 5 | | 9 |
| 318 | Profam clinics are a one stop centre for health needs and services | 1 | | 2 | | 3 | | 4 | | 5 | | 9 |
| **Quality of Care** | | | | | | | | | | | |  |
| 319 | Family planning services provided in public health facilities are of high quality | | 1 | | 2 | | 3 | | 4 | | 5 | 9 |
| 320 | Family planning services provided in Profam clinics are of high quality | | 1 | | 2 | | 3 | | 4 | | 5 | 9 |
| 321 | Providers at Profam clinics are friendly and helpful | | 1 | | 2 | | 3 | | 4 | | 5 | 9 |
| 322 | Health providers at Profam clinics attend to my questions and concerns very well | | 1 | | 2 | | 3 | | 4 | | 5 | 9 |
| **Social Norms** | | |  | |  | |  | |  | |  |  |
| 323 | In this community, many women use modern contraceptive methods of family planning | | 1 | | 2 | | 3 | | 4 | | 5 | 9 |
| 324 | In my community, men do not like their wives to use FP. | | 1 | | 2 | | 3 | | 4 | | 5 | 9 |
| 325 | In my community, religious leaders do not support the use of FP. | | 1 | | 2 | | 3 | | 4 | | 5 | 9 |
| 326 | Many women in my community use IUCD to limit/space births | | 1 | | 2 | | 3 | | 4 | | 5 | 9 |
| 327 | Many women in my community use implant to limit/space births. | | 1 | | 2 | | 3 | | 4 | | 5 | 9 |
| 328 | In my community, women found to be using family planning secretly can be punished, beaten, or divorced by their husbands. | | 1 | | 2 | | 3 | | 4 | | 5 | 9 |

| **ABILITY** | | |  |  |
| --- | --- | --- | --- | --- |
| **Knowledge** | | **TRUE** | **FALSE** | **DK** |
| 329 | Risk of infant death is higher if a woman gives birth too frequently/every year. | 1 | 0 | 9 |
| 330 | Risk of maternal death is higher if a woman gives birth too frequently/every year. | 1 | 0 | 9 |
| 331 | Spacing of children for 3 to 5 years between births reduces the risk of death of a mother and child. | 1 | 0 | 9 |
| 332 | A woman who uses contraception may not be able to have children later even after she stops | 1 | 0 | 9 |
| 333 | IUCDs can be used while breast feeding | 1 | 0 | 9 |
| 334 | Once the IUCD is removed, a woman can get pregnant again | 1 | 0 | 9 |
| 335 | IUCD is almost as effective as sterilization. | 1 | 0 | 9 |
| 336 | IUCD can protect women from STIs | 1 | 0 | 9 |

| ***Interviewer say:*** Please tell me if you “agree” or “disagree” Whenever I say ‘I’ am referring to ‘You’. Interviewer to probe if respondents “strongly agree” or “agree,” and if they “strongly disagree” or “disagree” | | | | | | | | |
| --- | --- | --- | --- | --- | --- | --- | --- | --- |
| **Social support** | | | | | | | | |
|  | | **Strongly Disagree** | **Somewhat**  **Disagree** | | **Neutral** | **Somewhat Agree** | **Strongly Agree** | **DK** |
| 337 | My friends encourage me to use IUCD | 1 | 2 | | 3 | 4 | 5 | 9 |
| 338 | My friends encourage me to use modern contraceptive methods. | 1 | 2 | | 3 | 4 | 5 | 9 |
| 339 | My partner encourages me to use modern family planning. | 1 | 2 | | 3 | 4 | 5 | 9 |
| 340 | My partner encourages me to use IUCD | 1 | 2 | | 3 | 4 | 5 | 9 |
| **Self efficacy** | | | | | | | | |
| 341 | I am embarrassed to get/ask about IUCD from a health facility | 1 | | 2 | 3 | 4 | 5 | 9 |
| 342 | I am capable of convincing my husband/partner to use family planning | 1 | | 2 | 3 | 4 | 5 | 9 |

| **MOTIVATION** | | | | | | | | |
| --- | --- | --- | --- | --- | --- | --- | --- | --- |
|  | | | **Strongly Disagree** | **Somewhat**  **Disagree** | **Neutral** | **Somewhat Agree** | **Strongly Agree** | **DK** |
| **Attitude** | | |  |  |  |  |  |  |
| 343 | | IUCD is a good method of family planning | 1 | 2 | 3 | 4 | 5 | 9 |
| 344 | | IUCDs are safe | 1 | 2 | 3 | 4 | 5 | 9 |
| 345 | | I would recommend an IUCD to friend | 1 | 2 | 3 | 4 | 5 | 9 |
| 346 | | Implants are safe | 1 | 2 | 3 | 4 | 5 | 9 |
| 347 | | IUCD is an effective contraceptive method for women like me (LF) | 1 | 2 | 3 | 4 | 5 | 9 |
| **Belief** | | |  |  |  |  |  |  |
| 348 | Using UCDs can result in cancer | | 1 | 2 | 3 | 4 | 5 | 9 |
| 349 | IUCDs can damage the womb | | 1 | 2 | 3 | 4 | 5 | 9 |
| 350 | Prolonged use of IUCDs can cause infertility | | 1 | 2 | 3 | 4 | 5 | 9 |
| 351 | The IUCD will “fall out” during hard physical labor. | | 1 | 2 | 3 | 4 | 5 | 9 |
| 352 | Using IUCDs reduces sexual pleasure | | 1 | 2 | 3 | 4 | 5 | 9 |
| 353 | Children born to a woman who used an IUCD can experience various health problems | | 1 | 2 | 3 | 4 | 5 | 9 |
| **Outcome expectation** | | |  |  |  |  |  |  |
| 354 | Implants are highly effective to prevent unwanted pregnancies. | | 1 | 2 | 3 | 4 | 5 | 9 |
| 355 | IUCDs are highly effective to prevent unwanted pregnancies. | | 1 | 2 | 3 | 4 | 5 | 9 |
| **Threat** | | |  |  |  |  |  |  |
| ***Severity*** | | |  |  |  |  |  |  |
| 356 | Unintended pregnancy is a major problem in my community. | | 1 | 2 | 3 | 4 | 5 | 9 |
| 357 | Having many children may result in financial hardship for families. | | 1 | 2 | 3 | 4 | 5 | 9 |
| ***Susceptibility*** | | |  |  |  |  |  | 5 |
| 358 | I am worried about unintended pregnancies. | | 1 | 2 | 3 | 4 | 5 | 9 |

**SECTION IV: EXPOSURE TO COMMUNICATIONS ABOUT FAMILY PLANNING**

COMPLETE THIS SECTION FOR EVERY ELIGIBLE WOMAN OF REPRODUCTIVE AGE (WRA)

***Interviewer: in this section, I would like to ask questions about what you have heard or seen about the different messages/communication about family planning***

|  | **Question** | | | | | **Responses** | | | **Code** | | **Skip** | |
| --- | --- | --- | --- | --- | --- | --- | --- | --- | --- | --- | --- | --- |
| Q401 | Have you seen /heard any communication/messages/activities about family planning? | | | | | Yes  No | | | 1  0 | | **If no go to 405** | |
| Q402 | Please describe the content of the message/ communication/ activities that you have heard/seen.  **Interviewer: Keep prompting until all known content or messages are mentioned** | | | | Use family planning  Space your children  Use IUCD  Use Implants  Use FP injectables  Use FP pills  Use female/male sterilization  Use long term family planning methods  Get family planning from a Profam facility  Get family planning for a good life/UHMG facility  Get family planning from a blue star facility  Get FP where you find the yellow flower sign  Others (specify)___________________ | | | | 1  1  1  1  1  1  1  1  1  1  1  1  1 | | 0  0  0  0  0  0  0  0  0  0  0  0  0 | |
| Q403 | Please describe the messages/ communications/activities you have seen or heard about family planning?  ***(Interviewer: Probe for more descriptions of communications heard or seen and Categorise whether PACE only, non PACE only or both)*** | | | | | PACE/Profam communication only  Non-PACE communication only  Both | | | 1  2  3 | |  | |
| Q404 | Where have you seen or heard any messages /communication/activities about family planning?  **Interviewer: Probe to get more sources** | **Profam/PACE Communications** | | | | **Y** | **N** | **Non- PACE** | | **Y** | | **N** |
|  |  | Profam provider  Other Health worker  Village health worker  Television  Radio  Phone voice /text messages  Wall branding  Poster  Sign posts  Branded T-shirt / Aprons  Drama / Videos  Mobile Mega phones  Static mega phones  Rig Truck  SMS  Women groups  Others (specify)__________________ | | | | 1  1  1  1  1  1  1  1  1  1  1  1  1  1  1  1  1 | 0  0  0  0  0  0  0  0  0  0  0  0  0  0  0  0  0 | Health worker  Village health worker  Television  Radio  Phone voice messages  Phone text messages  Wall branding  Poster  Sign posts  Branded T-shirt / Aprons  Drama / Videos  Mobile Mega phones  Static mega phones  Rig Truck  SMS  Women groups  Others (specify)_____________ | | 1  1  1  1  1  1  1  1  1  1  1  1  1  1  1  1  1 | | 0  0  0  0  0  0  0  0  0  0  0  0  0  0  0  0  0 |
| Q405 | In the last **6 months,** have you been visited by a village health worker and discussed about family planning services at Profam clinics? | | | | | Yes  Yes but not in last 6 months  Never been visited | | | 1  2  3 | | **If 3 go to 409** | |
| Q406 | In the last **6 months**, about how many times have you been visited by a village health worker to discuss about family planning? | | | | | ………………………….Times | | |  | |  | |
| Q407 | What was the main message of the session, according to you? | | | IUDs are safe  IUDs are effective  IUDs easy to use  Affordable  Others (specify)____________________________ | | | | | 1  2  3  4  96 | |  | |
| Q408 | How long was the discussion with the village health worker the last time you were visited? | | | | | Less than10 minutes  10-30 minutes  More than 30 minutes | | | 1  2  3 | |  | |
| Q409 | In the **last 6 months**, have you been visited by a village health worker and discussed about family planning services at blue star clinics | | | | | Yes  Yes but not in last 6 months  Never been visited | | | 1  2  3 | |  | |
| Q410 | In the **last 6 months,** have you been visited by a village health worker and discussed about family planning services at good life clinics | | | | | Yes  Yes but not in last 6 months  Never been visited | | | 1  2  3 | |  | |
| Q411 | In the last **12 months,** have you got/heard/seen any message or communication from someone who has ever got family planning services from a Profam facility? | | | | | Yes  No | | | 1  2 | | **If no skip to 413** | |
| Q411b | In the last **12 months,** about how many times have you got any message or communication from someone who has ever got family planning services from a Profam facility? | | | | | ………………………….Times | | |  | |  | |
| Q412 | How long was the discussion with that person who has ever got a family service from a Profam facility?  (***Ask about the most recent time the discussion was held***) | | | | | Less than10 minutes  10-30 minutes  More than 30 minutes | | | 1  2  3 | |  | |
| Q413 | In the last **6 months**, have you heard a **radio** communication about Profam? | | | | | Yes  No | | | 1  2 | | **If no skip to 414** | |
| Q413b | In the last **6 months**, about how many times have you heard a **radio** communication about Profam? | | | | | …………………Times | | |  | |  | |
| Q414 | Have you seen a Profam health facility in this sub county? ***(Ask about Parish for Kampala)*** | | Seen Profam facility within sub county only  Seen Profam facility elsewhere only  Seen Profam facility within and elsewhere  No, Never seen Profam facility anywhere | | | | | | 1  2  3  4 | |  | |
| Q415 | In the last **12 months**, have you visited a Profam health facility? | | | | | Yes  No  Never visited a Profam facility | | | 1  0  2 | |  | |
| Q416 | In the last **12 months** have you ever been informed by a Profam health provider to use IUDs for family planning? | | | | | Yes  No | | | 1  0 | |  | |
| Q417 | Which service have you ever got from the Profam facility  ***Multiple response*** | | | | | Family planning service  Cancer screening  Antenatal care  Delivery  Other maternal health services  Other health service  None  Never been to a Profam clinic  Other (Specify)_______________ | | | 1  2  3  4  5  6  7  8  96 | |  | |
| Q418 | Can you finish the phrase **Profam/do you know the Profam slogan……..**? | | | | | …Caring for you  Cannot complete phrase / don’t know  Other (Specify)______________ | | | 1  0  96 | |  | |
| Q419 | What does the Profam logo mean to you? ***(show Profam logo)***  ***Multiple response possible*** | | | | | Availability of family planning services  Manageable family  Quality family planning services  Don’t Know  Other (Specify)_______________ | | | 1  2  3  99  96 | |  | |
| Q420 | Have you ever purchased / received a voucher to go for family planning services in the **last 12 months**?  We still have SES questions, revise instruction to move to Q501 | | | | | Yes  No | | | 1  0 | | **If No, End Interview** | |
| Q421 | How much did you pay for the family planning voucher that you received? | | | | | Free  Ushs ___________ | | | 0  1 | |  | |
| Q422 | To which health facility were you referred to by the person who issued you the voucher? | | | | | Profam facility  Blue star facility  Good life facility  Other (Specify)______________ | | | 1  2  3  96 | |  | |

|  |  |  |  |  |
| --- | --- | --- | --- | --- |
| Q423 | Which service did you get from the facility where you were referred when issued a voucher? | Never used the voucher  Family planning method  Other health-related service  None  Not yet gone for service  Other (Specify) __________________ | 1  2  3  4  5  96 |  |

**SECTION V: HOUSEHOLD ASSETS AND SOCIO-ECONOMIC STATUS**

COMPLETE THIS SECTION FOR EVERY ELIGIBLE WOMAN OF REPRODUCTIVE AGE (WRA)

| **NO** | **QUESTION/FILTERS** | **RESPONSES** | | **CODE** | **SKIP** |
| --- | --- | --- | --- | --- | --- |
| **Q501** | What is the **main source** of drinking water for members of your household? | **PIPED WATER**  Piped into dwelling  Piped to yard/plot  Public tap/standpipe  **WATER FROM OPEN WELL /SPRING**  Open well/Spring in yard/plot  Open public well/spring  **WATER FROM PROTECTED WELL/SPRING**  Protected well/spring in yard/plot  Protected public well/spring  **WATER FROM BOREHOLE**  Borehole in yard/plot  Public borehole  **SURFACE WATER (RIVER/DAM)**  River/stream  Pond/Lake  Dam  Rain water  Tanker truck  Vendor  Bottled water  Other (specify)________________ | | 1  2  3  4  5  6  7  8  9  10  11  12  13  14  15  16  96 |  |
| **Q502** | What kind of toilet facility do members of your household usually use? | Flush toilet or pour toilet  VIP Latrine  Covered pit latrine no slab  Covered pit latrine with slab  Uncovered pit latrine no slab  Uncovered pit latrine with slab  Composting toilet  Bucket toilet  Ecosan  No facility/ bush/ field  Other (specify)________________ | | 1  2  3  4  5  6  7  8  9  10  96 |  |
| **Q503** | Does your household have:  ***READ ALL ANSWERS, MULTIPLE RESPONSES POSSIBLE.*** | **Possession** | **Yes** | **No** |  |
|  |  | a. Electricity  b. Radio  c. Cassette player  d. Television  e. Mobile phone  f. Fixed phone  g. Refrigerator  h. Table  i. Chair  j. Sofa set  k. Bed  l Cupboard  m. Clock | 1  1  1  1  1  1  1  1  1  1  1  1  1 | 0  0  0  0  0  0  0  0  0  0  0  0  0 |  |
| **Q504** | Does **any member** of your household own:  ***READ ALL ANSWERS, MULTIPLE RESPONSES POSSIBLE.*** | a. Bicycle  b. Motorcycle/ Scooter  c. Car/ truck  d. Boat with motor  e. Boat with no motor | 1  1  1  1  1 | 0  0  0  0  0 |  |
| **Q505** | What type of fuel does your household **mainly use** for cooking?    ***DON’T READ ANSWERS, SELECT ONE RESPONSE*** | Electricity  LPG/Natural gas  Biogas  Kerosene /paraffin  Charcoal  Firewood  Straw/shrub/grass  Animal dung  No food cooked in household  Other (**Specify**)__________ | 1  2  3  4  5  6  7  8  9  96 |  | |
| **Q506** | What is the **main material** of the floor of your main house?  ***DON’T READ ANSWERS, SELECT ONE RESPONSE*** | **NATURAL FLOOR**  Earth / sand  Earth and Dung  **FINISHED FLOOR**  Parquet or polished wood  Mosaic or tiles  Bricks  Cement  Stones  Other (**Specify**)__________ | 1  2  3  4  5  6  7  96 |  | |
| **Q507** | What is the **main material** of the roof of your main house?  ***DON’T READ ANSWERS, SELECT ONE RESPONSE*** | **NATURAL ROOF**  Thatched  Mud  **RUDIMENTARY ROOF**  Wood/ planks  Iron sheets  Asbestos  Tiles  Tin  Cement  Other (**Specify**)__________ | 1  2  3  4  5  6  7  8  96 |  | |
| **Q508** | What is the **main material** of the walls of your main house at home?  ***DON’T READ ANSWERS, SELECT ONE RESPONSE*** | **NATURAL WALL**  Thatched/straw  **RUDIMENTARY WALL**  Mud and poles  Un burnt bricks  Un burnt bricks with plaster  Burnt bricks with mud  **FINISHED WALLS**  Cement blocks  Stone  Timber  Burnt bricks with cement  Other (**Specify**)__________ | 1  2  3  4  5  6  6  7  8  9  96 |  | |
| **Q509** | How many rooms in your household are **used for sleeping**? | **ROOMS** |  |  | |

**This is the end of our interview. Thank you for your time.**
